# Purification, characterization, and structural elucidation of serralysin-like alkaline metalloprotease from a novel source

**DOI:** 10.1186/s43141-019-0002-7

**Published:** 2019-09-23

**Authors:** Swathi Nageswara, Girijasankar Guntuku, Bhagya Lakshmi Yakkali

**Affiliations:** 1 A.U. College of Pharmaceutical Sciences, Andhra University, Visakhapatnam, Andhra Pradesh 530003 India; 2 Srikakulam, India

**Keywords:** Purification, Characterization, *Streptomyces hydrogenans* var. MGS13, Metalloendopeptidases, Partial amino acid sequence, Serralysin

## Abstract

**Background:**

Serratiopeptidase is an alkaline metalloendopeptidase, which acquired wide significance because of its therapeutic applications. The present study was undertaken for purification, characterization, and structural elucidation of serratiopeptidase produced from *Streptomyces hydrogenans* var. MGS13.

**Result:**

The crude enzyme was purified by precipitating with ammonium sulfate, dialysis, and Sephadex gel filtration, resulting in 34% recovery with a 12% purification fold. The purified enzyme S.AMP13 was spotted as a single clear hydrolytic band on casein zymogram and whose molecular weight was found to be 32 kDa by SDS-PAGE. The inhibitor and stability studies revealed that this enzyme is metalloprotease, thermostable, and alkaline in nature. The maximum serratiopeptidase activity was observed at 37 °C and pH 9.0. The partial amino acid sequence of the purified enzyme S.AMP13 by LC-MS/MS analysis shows the closest sequence similarities with previously reported alkaline metalloendopeptidases. The amino acid sequence alignment of S.AMP13 shared a conserved C-terminus region with peptidase-M10 serralysin superfamily at amino acid positions 128–147, i.e., ANLSTRATDTVYGFNSTAGR revealed that this enzyme is a serralysin-like protease. The kinetic studies of the purified enzyme revealed a *K*_*m*_ of 1 mg/mL for its substrate casein and *V*_max_ of 319 U/mL/min. The 3D structure of the purified enzyme was modeled by using SWISS-MODEL, and the quality of the structure was authenticated by assessing the Ramachandran plot using PROCHECK server, which suggested that the enzyme was stable with good quality.

**Conclusion:**

Inhibitor, stability, electrophoretic, and bioinformatic studies suggested that the purified enzyme obtained from *S. hydrogenans* var. MGS13 is a serralysin-like protease.

**Electronic supplementary material:**

The online version of this article (10.1186/s43141-019-0002-7) contains supplementary material, which is available to authorized users.

## Background

Peptidases are hydrolases that catalyze the hydrolysis of peptide and iso-peptide bonds that join amino acids within proteins, and based on the catalytic mechanism, peptidases are classified as metalloserine, aspartic, cysteine, and threonine [1]. Among these proteases, metalloproteases represent the largest class of hydrolases that usually contain divalent metal ions at an active site which plays an important role in proteolysis.

Serratiopeptidase (EC 3.4.24.40) also known as serrapeptase and serralysin, which is a type of metalloendopeptidase, possess anti-edemic, anti-inflammatory analgesic, fibrinolytic, and anti-atherosclerotic properties and have been directly employed in clinical therapy in regulation of inflammation and pain, and furthermore, serrapeptase is even being used as a health supplement to protect the heart from atherosclerosis, which was achieved by degradation of atherosclerotic plaque and fibrin on the inside of arteries [2]. The anti-inflammatory and analgesic activity of serratiopeptidase is achieved by degrading the inflammation-causing amino acid derivatives such as histamine, serotonin, and bradykinin [3]. Serratiopeptidase is an alkaline metallopeptidase originally isolated from *Serratia marcescens*; later homolog of this enzyme was also reported from some genera of gram-negative and positive bacteria, such as *Pseudomonas aeruginosa*, *Proteus mirabilis*, *Erwinia chrysanthemi* [4, 5], *Xenorhabdus* [6, 7], *Deinococcus radiodurans* [8], and *Bacillus subtilis* [9]. Serralysin contains one atom of zinc per molecule as an essential element indicating that serralysin family belongs to the metzincin class of proteases according to the MEROPS database [10]. Presently, serratiopeptidase obtained from *Serratia E-15*, an opportunistic pathogen, is being used in therapy for inflammation; due to its pathogenicity, the enzyme has been reported to cause lung and corneal damage [11]. This problem has necessitated screening of new microbial strains for producing the serratiopeptidase from novel sources with better therapeutic potential and desirable characters.

*Streptomyces* has gained attention in the present decade due to the discovery of various fibrinolytic enzymes (plasmin-like [12], serine-like [13], and chymotrypsin-like serine-metallo fibrinolytic enzymes) [14]. Furthermore, Jyothi et al. earlier isolated a serratiopeptidase-producing organism in our laboratory, identified as *Streptomyces hydrogenans* var. MGS13 [15], which was used for the production of serratiopeptidase by submerged fermentation technology [16]. Increasing attention is being given to the production of microbial metabolites by solid-state fermentation (SSF) as it has many advantages over submerged fermentation. Hence, in the present study, SSF was selected for the production of serratiopeptidase from *S. hydrogenans* var. MGS13. Purification of enzymes is a challenging and essential step for identifying the enzymes with their structure-functional properties, and the role of purification is to achieve the utmost possible purity and yield of the desired enzyme with the maximum catalytic activity. The combination of two-dimensional (2D) gel electrophoresis and peptide mass fingerprinting analysis by mass spectrometry is a widely used strategy in proteomics study over traditional method where proteins were identified by de novo sequencing using automated Edman degradation method [17].

The present work throws light on the purification and characterization of serralysin-like enzyme with respect to the peptide mapping by LC-MS/MS for its partial amino acid sequence alignment, followed by structural elucidation using various bioinformatic tools.

## Methods

### Design of the study

The new strain *Streptomyces hydrogenans* var. MGS13 isolated from Koringa mangrove soil was used in the present study for the production of serratiopeptidase by solid-state fermentation. The enzyme was purified by employing ammonium sulfate precipitation, followed by dialysis and gel filtration. Further characterization was done by performing stability and inhibitor studies. Finally, a combination of 2D gel electrophoresis, mass spectrometry, and bioinformatic tools was used for structural elucidation of purified serratiopeptidase.

### Serratiopeptidase production

Serratiopeptidase was obtained from *S. hydrogenans* var. MGS13 using optimized medium composed of horse gram (4.8 g; particle size 600–250 μm), soya bean 1.1%, initial moisture content 42% and sterilized at 121 °C at 15 lbs pressure for 20 min. After cooling, 1.2 mL of inoculum was added and incubated at 28 °C for 4 days then the crude enzyme was obtained by extracting with sodium borate buffer (pH 9.0).

### Determination of serratiopeptidase activity and protein content

Quantitative estimation of serratiopeptidase activity was determined according to IP 2010 [18]. One unit of serratiopeptidase was defined as the amount of enzyme required to liberate 1 μm of free tyrosine per min under standard assay conditions. Protein content was quantified according to the Lowry method [19] using bovine serum albumin as a protein standard.

### Purification of serratiopeptidase produced from *S. hydrogenans* var. MGS13

An attempt was made to decide the optimal concentration required for the precipitation of the enzyme. For this purpose, various concentrations of ammonium sulfate were supplemented to the supernatant to attain 40–80% saturation. The precipitated proteins were redissolved in minimum quantity of sodium borate hydrochloric acid buffer pH 9.0 and dialyzed using dialysis membrane-50 (HiMedia—cutoff value 12 kDa) against the same borate buffer overnight at 4 °C. Then, the sample was concentrated, desalted, and loaded on the Sephadex G-100 column (30 × 1.8 cm) for purification. The enzyme fractions were eluted using sodium borate buffer pH 9.0 with a flow rate of 0.3 mL/min. The fractions eluted from the gel filtration column were tested for serratiopeptidase activity and total protein content. The fractions having enzyme activity were pooled, concentrated, and used as a purified sample for all the characterization studies. The recovery of enzyme and purification fold was calculated in terms of specific activity.

### Electrophoretic analysis

The molecular weight of the purified protease was analyzed by SDS-PAGE according to the Laemmli method [20] using 15% polyacrylamide resolving gel. The mass was ascertained by comparing with pre-stained known molecular weight markers ranging from 10 to 245 kDa, HiMedia.

Casein zymography of purified serratiopeptidase was performed according to the Garcia-Carreno method [21] using casein (0.2% *w*/*v*) as a substrate in 15% resolving gel. The sample was dissolved in non-reducing SDS-loading buffer without heating. Subsequent to electrophoresis, the gel was kept for 1 h for incubation in 100 mL of sodium borate buffer pH 9.0 containing 2.5% (*v*/*v*) Triton X-100 to remove SDS and thoroughly rinsed with double distilled water to remove Triton X-100. Then, the gel was incubated in a buffer for an hour and later stained with 0.025% Coomassie Brilliant Blue R-250 and de-stained.

### Effect of pH on serratiopeptidase stability and activity

The influence of pH on serratiopeptidase stability was determined by exposing the purified serratiopeptidase to various pH buffers of 50 mM concentration at 37 °C for 1 h, and the residual serratiopeptidase activity was measured. The buffers used for this study were phosphate buffer pH 4.0, 5.0, 6.0, 7.0, and 8.0; borate buffer pH 9.0 and 10.0; and glycine buffer 11.0. Optimum pH for serratiopeptidase activity was determined by preparing the substrate (casein) in the above buffers, and serratiopeptidase activity was measured as mentioned earlier.

### Effect of temperature on serratiopeptidase stability and activity

The influence of temperature on serratiopeptidase stability was determined by incubating the purified protease at various temperatures 20 °C, 28 °C, 37 °C, 50 °C, and 60 °C for 1 h in sodium borate buffer at pH 9.0, followed by serratiopeptidase assay, whereas, the optimum temperature for serratiopeptidase activity was measured by performing the assay at diverse temperatures ranging from 20 °C, 28 °C, 37 °C, 50 °C, and 60 °C for 30 min in sodium borate buffer of pH 9.0.

### Effect of inhibitors on enzyme activity

The influence of chelating agents such as ethylene diamine tetra acetic acid (EDTA) and phenyl methyl sulfonyl fluoride (PMSF) on the activity of serratiopeptidase was determined by incubating the purified enzyme with 1 mM, 5 mM, and 10 mM concentrations of EDTA and PMSF solutions for 30 min at 37 °C, and the comparative activities were obtained by performing serratiopeptidase assay. The activity of the control (purified enzyme without any inhibitor) was determined and considered as 100%. The influence of chelating agents on the enzyme activity was also determined by casein clearing zone technique [22]. The purified enzyme S.AMP13 was incubated with inhibitors at concentrations of 1 mM, 5 mM, and 10 mM for 30 min at 37 °C and introduced into wells of casein agar plate after 24 h incubation; the activities were assessed by measuring the clearing zone.

### Effect of metal ions

The serratiopeptidase activity was assessed before and after the inactivation of metal ions from purified protease using 10 mM EDTA. In order to study the influence of metal ions on enzyme activity before chelation, the purified enzyme (1 mg/mL) was incubated for a time interval of 1 h at 37 °C with different metal ions at a concentration of 5 mM of Mg^+2^, Zn^+2^, Ca^+2^, Cu^+2^, Na^+^, and K^+^ in 50 mM of sodium borate buffer pH 9.0, and serratiopeptidase activity was determined as described earlier. For analyzing the metal ion effect after chelation, the purified enzyme was incubated with 10 mM EDTA, followed by incubation with different metal ions in sodium borate buffer 50 mM (pH 9.0) at 37 °C for 1 h, and the samples were evaluated by performing the serratiopeptidase assay. The activity of purified serratiopeptidase in a buffer without a chelating agent and metal ions (control) was assessed.

### Determination of kinetic parameters

The kinetic constants of the purified enzyme were determined by measuring the serratiopeptidase activity at different casein (substrate) concentrations (1.2 to 9.8 mg/mL). The *K*_*m*_ and *V*_max_ values were determined using the Michaelis-Menten graph, and the Lineweaver-Burk double-reciprocal graph was plotted with calculated values.

### Analysis of amino acid sequence

The purified enzyme was excised from the SDS-PAGE gel as a single protein band and named as S.AMP13 and dehydrated. After drying, the gel pieces were reduced with 10 mM dithiothreitol (DTT) in 100 mM ammonium bicarbonate, incubated at 56 °C for an hour and alkylated by incubating with iodoacetamide for 45 min at room temperature, then digested by incubating with trypsin solution overnight at 37 °C. The resulting tryptic digested peptides were extracted, and the supernatant was dispensed into an autosampler vial for peptide analysis by LC-MS. The tryptic digested peptides of 10-μL sample were injected in C_18_ UPLC column (75 μm × 150 mm), 1.7-μm particle size for the separation of peptides using a Water ACQUITY UPLC system, followed by analysis on the Q-TOF (model—Synapt G2) instrument for MS and MSMS. The column was eluted with a flow rate of 0.3 mL/min using buffers A (0.1% by volume formic acid (FA) in water) and B (0.1% by volume formic acid in acetonitrile (ACN)). The raw data was processed by Mass Lynx 4.1 Waters, peptide editor software, to get the complete integrated sequence of the sample. The individual peptide MSMS spectra were matched to the database sequence for amino acid sequence, and protein identification was assigned by searching a Swiss-Prot database containing all known alkaline metalloprotease from *Streptomyces* and other bacterial sources based on ProteinLynx Global SERVER (PLGS) score software, Waters. The obtained protein sequence was aligned with the similar proteins using “Clustal X2” [23] for the construction of pairwise sequence alignment from the sequence set. A dendrogram (guide tree) was constructed to analyze the phylogenetic relation of the newly identified protein by neighbor-joining (NJ) statistical method using MEGA 6.06 (Molecular Evolutionary Genetics Analysis) software. Conserved domain and motif in the sequence were identified using the conserved domain database of NCBI (https://www.ncbi.nlm.nih.gov/Structure/cdd/wrpsb.cgi) and motif scan (http://myhits.isb-sib.ch/cgibin/motif_scan).

### Prediction of structure

SWISS-MODEL server (http://swissmodel.expasy.org/interactive) was used to predict the 3D structure of the designed protein based on homology or comparative modeling, and the templates with the highest quality have been selected for model building. The best model was selected based on the QMEAN score. The computed model was structurally validated and analyzed by using PROCHECK of the PDBsum server, and the secondary structure and functions of protease were predicted using ProFunc server.

## Results

### Purification of serratiopeptidase

Serratiopeptidase produced by *S. hydrogenans* var. MGS13 was partially purified by precipitating the supernatant with different concentrations of ammonium sulfate to attain a maximum saturation. Among them, 50% saturation level yielded a maximum recovery of 52% partially purified protease suggesting 7% fold purification. The precipitated enzyme was resuspended in sodium borate hydrochloric acid buffer pH 9.0 and further dialyzed against the same buffer using 12-kDa cutoff membrane. This step yielded 9.7% fold purification with a specific enzyme activity of 40.3 units/mg of protein. Then, the partially purified enzyme was applied on Sephadex G-100 column for filtration, and active fractions showing serratiopeptidase activity were pooled. The specific activity of the final enzyme preparation was 50 U/mg of protein (Table 1). Overall, 12% fold purification and recovery of 34% yield were obtained at the end of purification steps.

**Table 1:** Summary of the purification of protease from *S. hydrogenans* var. MGS13

Purification stages	Total protein (mg)	Total enzyme activity units	Specific activity (U/mg)	Purification fold	Recovery (%)
Cell-free supernatant	12,460	51,670	4	1	100
50% ammonium sulfate precipitation	840	27,230	30	7	52
Dialyzed sample	770	22,200	40	9.7	43
Sephadex G-100	370.25	17,500	50	12	34

### Electrophoresis techniques

**Fig. 1:**
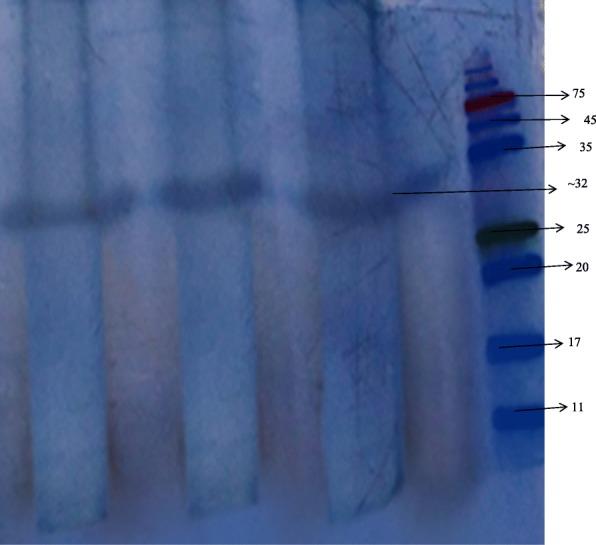
SDS-PAGE of purified serratiopeptidase. The molecular mass of purified serratiopeptidase was determined by comparing with protein markers

**Fig. 2:**
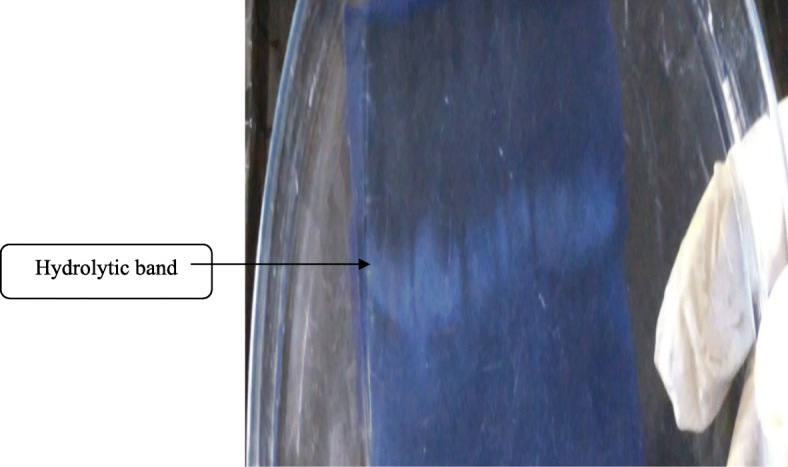
Casein zymogram of purified serratiopeptidase. Purified serratiopeptidase showing a clear hydrolytic band on casein polymerized gel

The observed single band in SDS-PAGE shows that the purified enzyme is homogenous with a molecular mass of 32 kDa (Fig. 1). Casein zymography also exhibited a single clear hydrolyzed band indicating that the purified protein is a “proteolytic enzyme” (Fig. 2).

### Enzyme characterization

#### Effect of pH on serratiopeptidase activity and stability

**Fig. 3:**
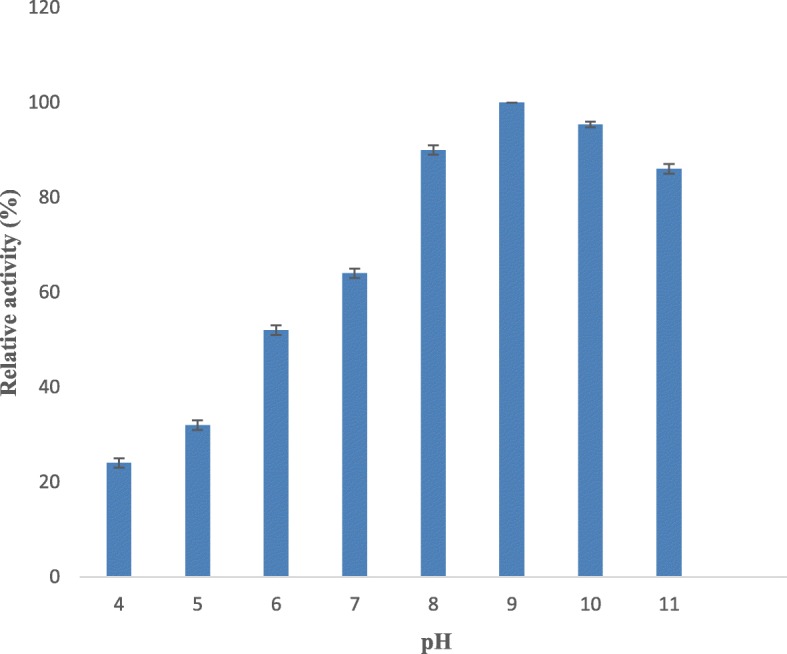
Effect of pH on serratiopeptidase activity. The enzyme activity was measured at various pH (4 to 11) using a standard assay method. Each value represents the mean ± SD for three determinations

Comparative activities of serratiopeptidase in various pH buffers have been measured, and maximum activity exhibited by the protease in buffer with pH 9.0 has been considered as 100% (Fig. 3). A very low activity (24%) was noted at pH 4.0, followed by pH 5.0 (32%), 6.0 (52%), and 7.0 (64%) which demonstrates that the enzyme is neither acidic nor neutral protease.

**Fig. 4:**
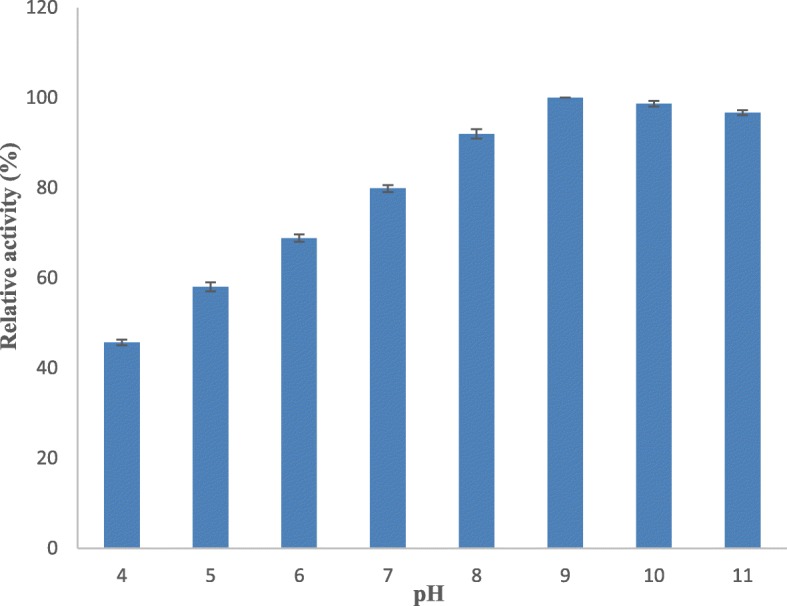
Effect of pH on serratiopeptidase stability. The enzyme stability was measured at various pH (4 to 11) using a standard assay method. Each value represents the mean ± SD for three determinations

Figure 4 shows the residual activities of serratiopeptidase in different pH buffers, and the purified protease of *S. hydrogenans* var. MGS13 showed greater than 50% serratiopeptidase activity at pH 7.0–10.0 after 1-h incubation period; the activity was substantially reduced below pH 7.0 and remains constant over 10.0.

#### Effect of temperature on serratiopeptidase activity and stability

**Fig. 5:**
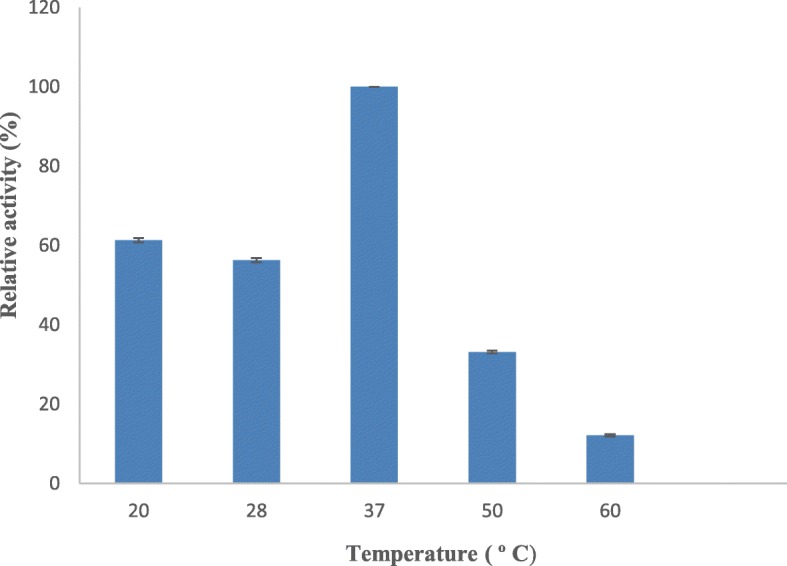
Effect of temperature on serratiopeptidase activity. The enzyme activity was measured at different temperatures (20 °C, 28 °C, 37 °C, 50 °C, and 60 °C) using a standard assay method. Each value represents the mean ± SD for three determinations

Comparative activities of serratiopeptidase at different temperatures have been measured, and maximum activity showed by the protease at 37 °C has been considered as 100% (Fig. 5). At 28 °C and 20 °C, the observed comparative activities were found to be 70% and 61%, respectively, and very low comparative activities of 33% and 12% were observed at 50 °C and 60 °C, respectively.

**Fig. 6:**
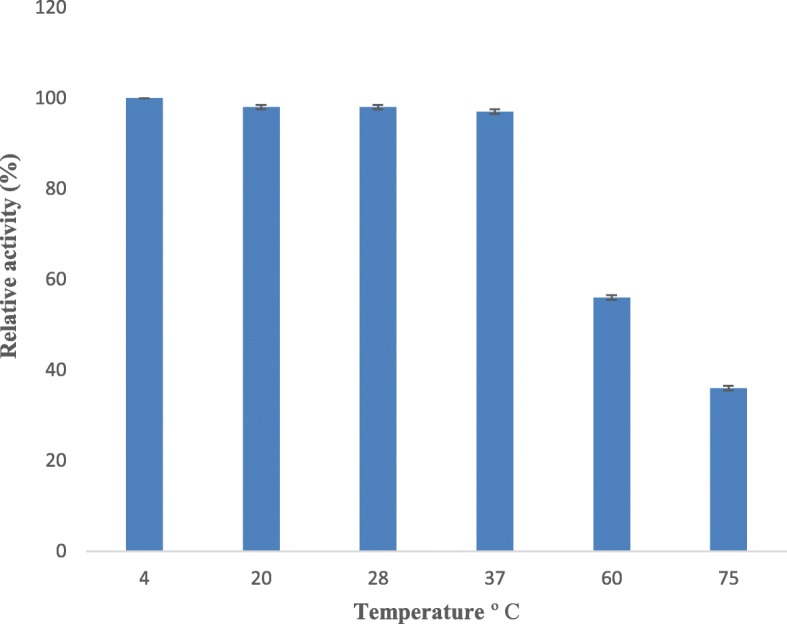
Effect of temperature on serratiopeptidase stability. The enzyme stability was measured at different temperatures ( 4 °C, 20 °C, 28 °C, 37 °C, 50 °C, and 60 °C ) using a standard assay method. Each value represents the mean ± SD for three determinations

Figure 6 shows the residual activities of protease obtained from *S. hydrogenans* var. MGS13 at different temperatures, and the serratiopeptidase activity prior to the incubation was evaluated and maximum activity was considered as “cent percent.” The purified protease was stable at 4 °C, and 56% residual activity (Fig. 6) was observed at 60 °C even after 30 min incubation.

#### Effect of inhibitors

**Fig. 7:**
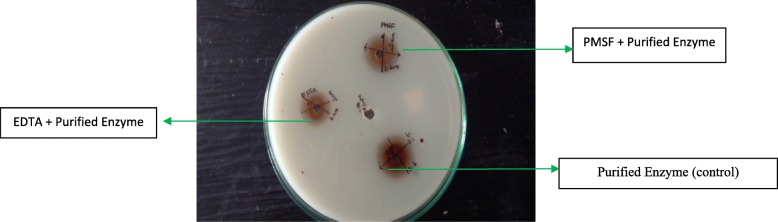
Effect of various inhibitors on purified protease. The inhibitors’ role was determined by measuring the casein hydrolytic zone after the pre-incubation of the enzyme with inhibitors. Control, purified enzyme without inhibitors; EDTA, ethylene diamine tetra acetic acid; PMSF, phenyl methyl sulfonyl fluoride

Serratiopeptidase activity shown by control (enzyme without inhibitors) was considered as 100% (Table 2), and activities after incubation with different inhibitors have been expressed relative to the control. The maximum inhibition of serratiopeptidase activity was noticed with 10 mM EDTA. Inhibitor studies by casein clear zone method also suggested that it is a metalloprotease, where a small clear zone (2 cm) was observed with 5 mM EDTA when compared to that of control (without inhibitor) and PMSF. The concentrations and zone diameters are shown in Fig. 7 and Table 3

**Table 2:** Effect of inhibitors on serratiopeptidase activity

Sample	Concentration (mM)	Serratiopeptidase activity
Control	–	100%
EDTA	1	55% ± 0.3
	5	42% ± 0.2
	10	39% ± 0.4
PMSF	1	80% ± 0.5
	5	72% ± 0.6
	10	70% ± 0.5

Each value represents the mean ± SD for three determinations

**Table 3:** Effect of various inhibitors on purified protease

Inhibitors	Concentration (mM)	Average of zone diameter (cm)
Control	–	2.6
PMSF	5	2.6
EDTA	5	2.0

*EDTA* ethylene diamine tetra acetic acid, *PMSF* phenyl methyl sulfonyl fluoride

#### Effect of metal ions on enzyme activity before and after chelation with EDTA

The role of metal ion on the catalytic activity of the enzyme was reckoned by adding 10 mM concentration of metal ion to the reaction mixture. At first, these investigations were carried out to know the role of the added metal ion in the regulation of serratiopeptidase activity. As a control, serratiopeptidase activity in the absence of these metal ions was considered as 100%, and any variation noticed due to the existence of these metal ions was considered as a metal ion-mediated activity. The data indicated that maximum serratiopeptidase activity of 138% was observed with Ca^2+^ followed by Zn^2+^, Na^+^, and K^+^ whereas the presence of metals like Mg^2+^ and Cu^2+^ in the reaction mixture retarded the catalytic activity.

The specific role of metal ion on the catalytic activity of purified protease obtained from *S. hydrogenans* var. MGS13 was ascertained by incubating the enzyme with 10 mM EDTA for chelation and followed by measuring the enzyme activity profile by supplementation of different selected metal ions at a concentration of 5 mM. Serratiopeptidase activity without the addition of EDTA and any metal salt was considered as 100% (control), and alteration observed after the addition of metal ion was considered as metal ion effect. In Table 4, it is conspicuous that the addition of EDTA resulted in a drastic reduction of serratiopeptidase activity and it was observed to be 12% after chelation with 10 mM EDTA, and the addition of metal ion to the same reaction mixture resulted in recovering the activity but this reactivation was metal ion-specific. A maximum of 68% enzyme activity was recovered with Ca^2+^ followed by Zn^2+^ (40%), Na^+^ (36%), and K^+^ (27%), while less than 20% activity was recovered with other metal ions such as Mg^2+^ (18%) and Cu^2+^ (19%)

**Table 4:** Effect of metal ions on serratiopeptidase activity

Metal ions (5 mM)	Relative activity before chelation and incubation with metal ions (%)	Regaining enzyme activity after chelation with EDTA and incubation with metal ions (%)
Control	100	–
Enzyme + EDTA (10 mM)	–	12 ± 0.8
Ca^2+^	138 ± 1.7	68 ± 1.6
Mg^2+^	92 ± 0.9	18 ± 1.0
Zn^2+^	109 ± 1.5	40 ± 1.4
Cu^2+^	98 ± 1.1	19 ± 1.0
Na^+^	105 ± 1.4	36 ± 1.3
K^+^	102 ± 1.3	27 ± 1.1

Each value represents the mean ± SD for three determinations

### Enzyme kinetic studies

**Fig. 8:**
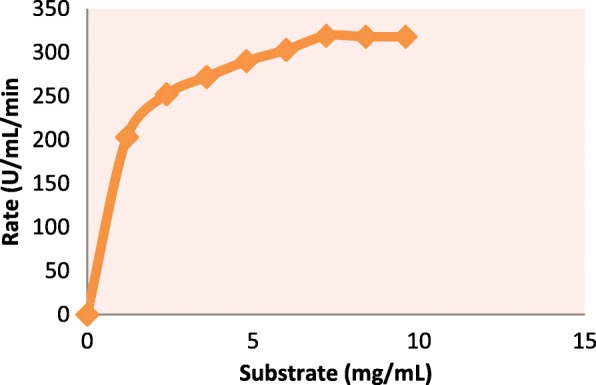
Michaelis-Menten plot of purified serratiopeptidase

**Fig. 9:**
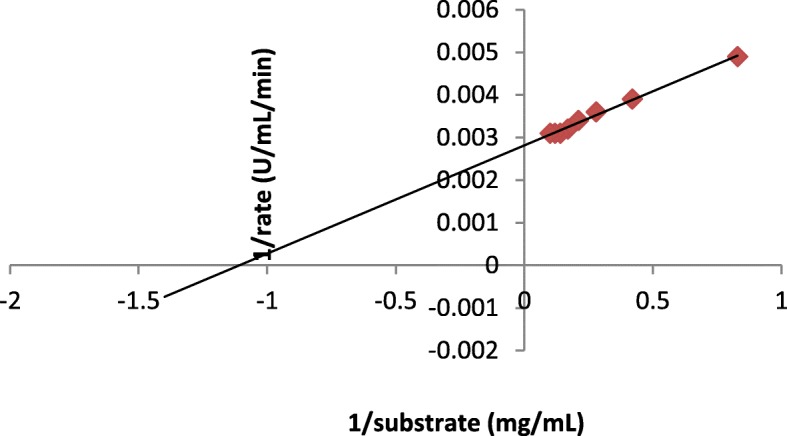
Lineweaver-Burk plot of purified serratiopeptidase

The effect of changing the substrate concentration on serratiopeptidase activity revealed that it follows Michelis-Menten curve (Fig. 8). It was noticed that this protease showed *K*_*m*_ of 1 mg/mL for its substrate casein and *V*_max_ of 319 U/mL/min. The Lineweaver-Burk(Fig. 9) double graph was plotted with the reciprocal of reaction velocity (1/*v*) as a function of reciprocal of substrate concentration (1/*S*).

### Peptide mapping by mass spectrometry

**Fig. 10:**
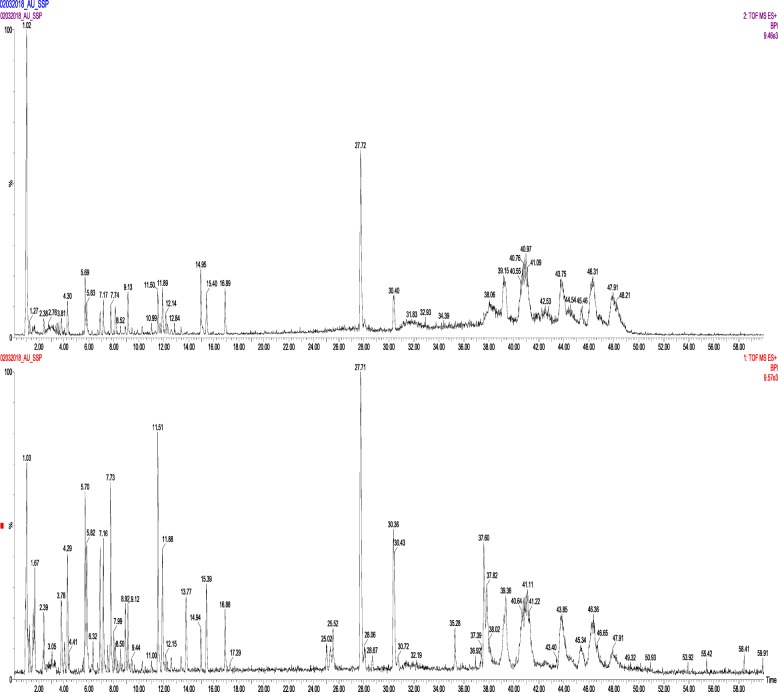
QTOF-MS of base peak intensity chromatograms of S.AMP13 from *S. hydrogenans* var. MGS13

The concerned protein of interest was isolated typically by SDS-PAGE and digested with trypsin to generate peptides further separated. The base peak intensities of each peptide fragments (Fig. 10) were analyzed by using LC-MS/MS, and the integrated sequence of the sample was obtained by processing with MassLynx 4.1 Waters, peptide editor software. The individual peptide MSMS spectra were matched to the database sequence for amino acid sequence, and protein S.AMP13 was confidently identified as alkaline metalloprotease based on ProteinLynx Global SERVER software WATERS (licensed software). The false discovery rate for the identification of peptides is 4%. The six trypsin-digested peptide fragment sequences of S.AMP13 matched with 33.7-kDa alkaline metalloendoprotease of *Pseudomonas syringae pv. maculicola* of Swiss-Prot database (KPB92383.1) with a sequence coverage of 46% and PLGS score of 139 (Table 5).

**Table 5:** Peptide matching of S.AMP13 with alkaline metalloprotease from *Pseudomonas syringae pv.maculicola* (Accession no. KPB92383.1)

Positions	Peptide sequence	Peptide M.W (Da)
3–31	VKENAAIQLSAATSTSFDQINTFAHEYDR	3227.56
5–54	ENAAIQLSAATSTSFDQINTFAHEYDRGGNLTINGKPSYSVDQAANFILR	5416.647
55–97	DDAAWADRDGNGTINLTYTFLTAKPAGFNNALGTFSAFNAQQK	4593.207
205–213	DATYAEDTR	1041.448
233–251	GGAPSYSSAPLLDDIAAVQQLYGANLSTR	2935.48
262–275	ATDTVYGFNSTAGR	1459.681

**Fig. 11:**
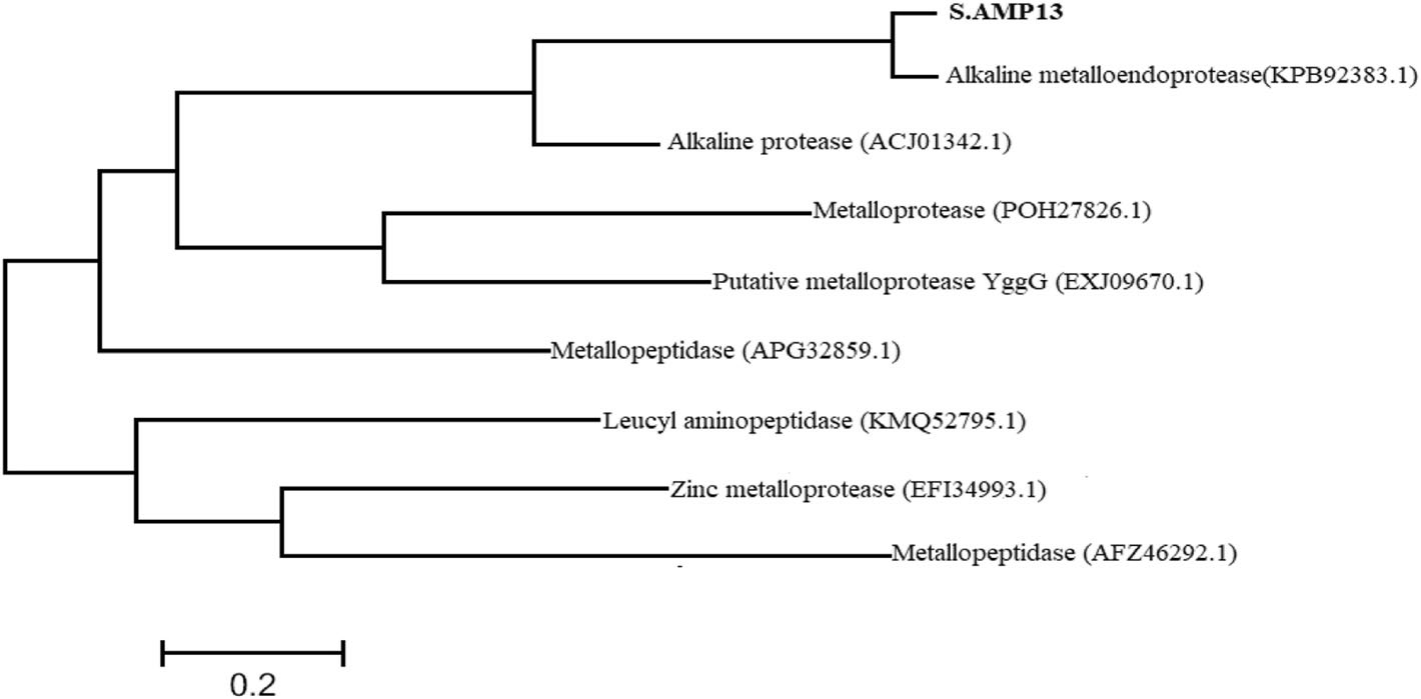
Phylogenetic tree analysis of S.AMP13 with other metalloproteases

Phylogenetic (Fig. 11) analysis of predicted protease S.AMP13 showed the relatedness towards the alkaline metalloendoprotease of *Pseudomonas* (Accession no. KPB92383.1) as they formed a single cluster.

**Fig. 12:**
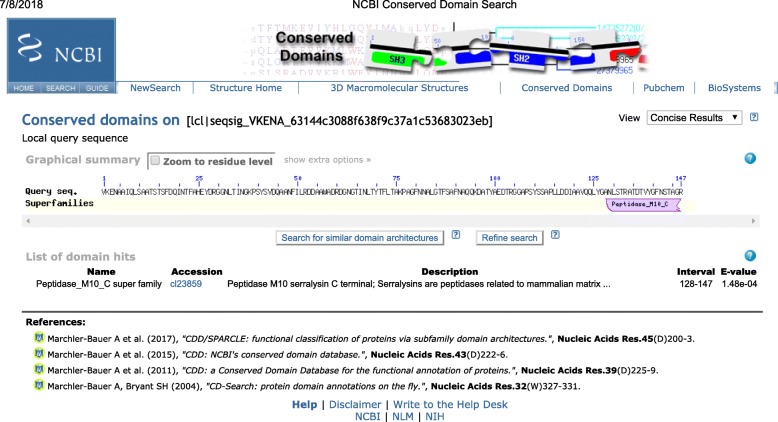
Conserved domains of S.AMP13

**Fig. 13:**
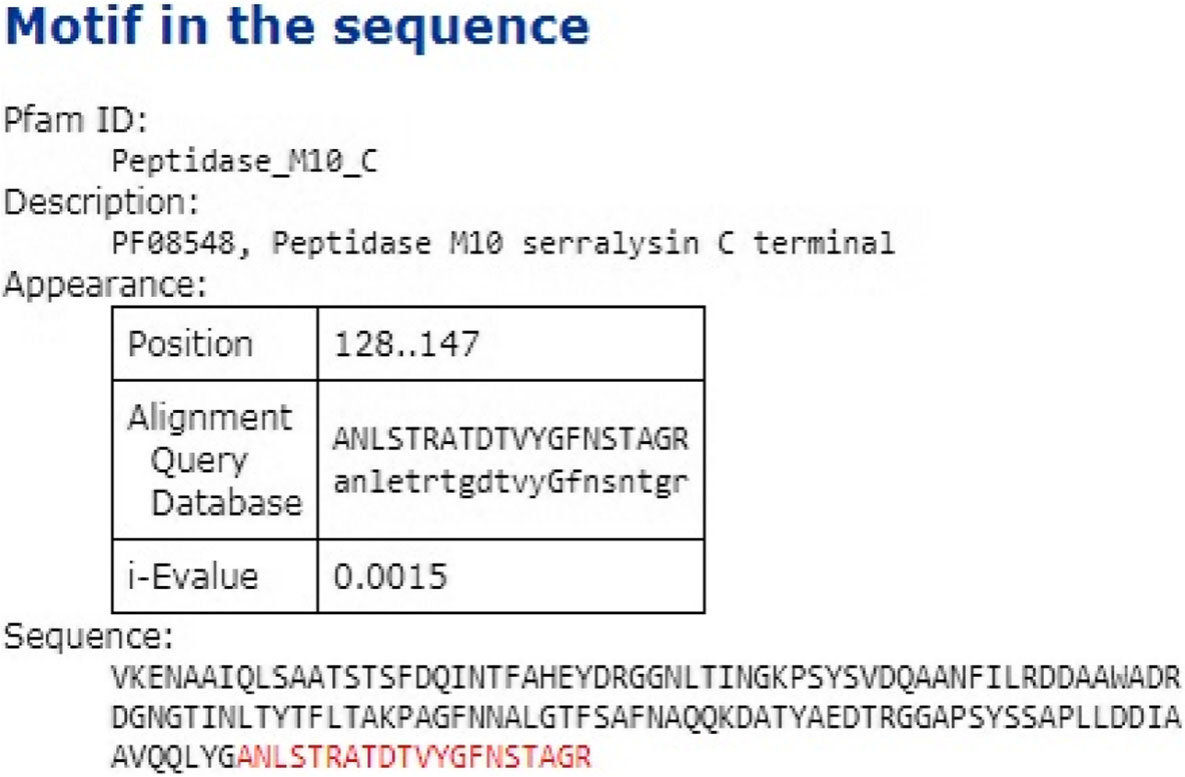
Motif matches of S.AMP13

The partial amino acid sequence of purified protease (S.AMP13) shared a conserved region with superfamily peptidase M10-C terminal at amino acid positions at 128–147, i.e., ANLSTRATDTVYGFNSTAGR indicating that this enzyme (S.AMP 13) might be a serralysin-like metalloprotease as shown in Figs. 12 and 13.

**Fig. 14:**
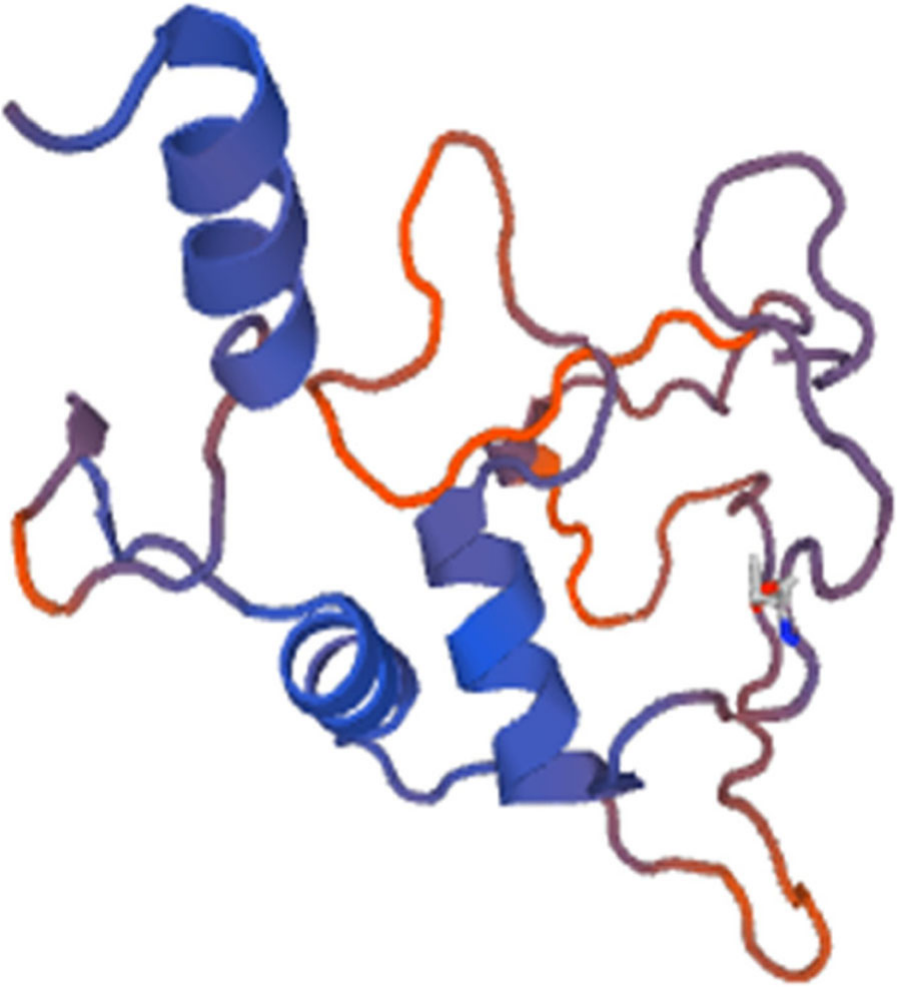
3D model of purified protease S.AMP13 by using SWISS-MODEL

**Fig. 15:**
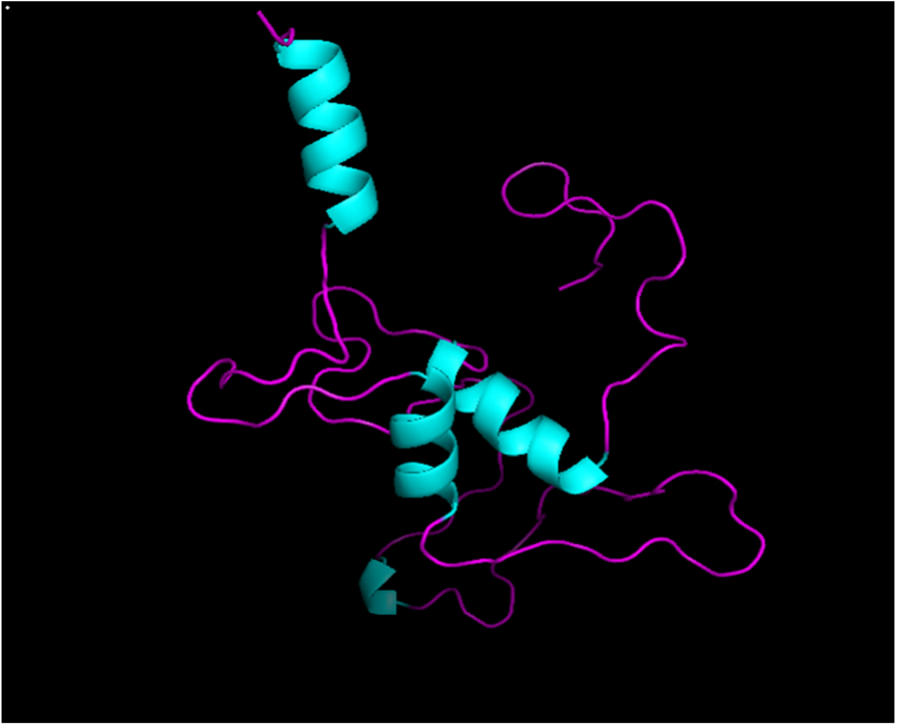
Visualization of the 3D structure of S.AMP13 by using PyMOL Molecular Graphics System

**Fig. 16:**
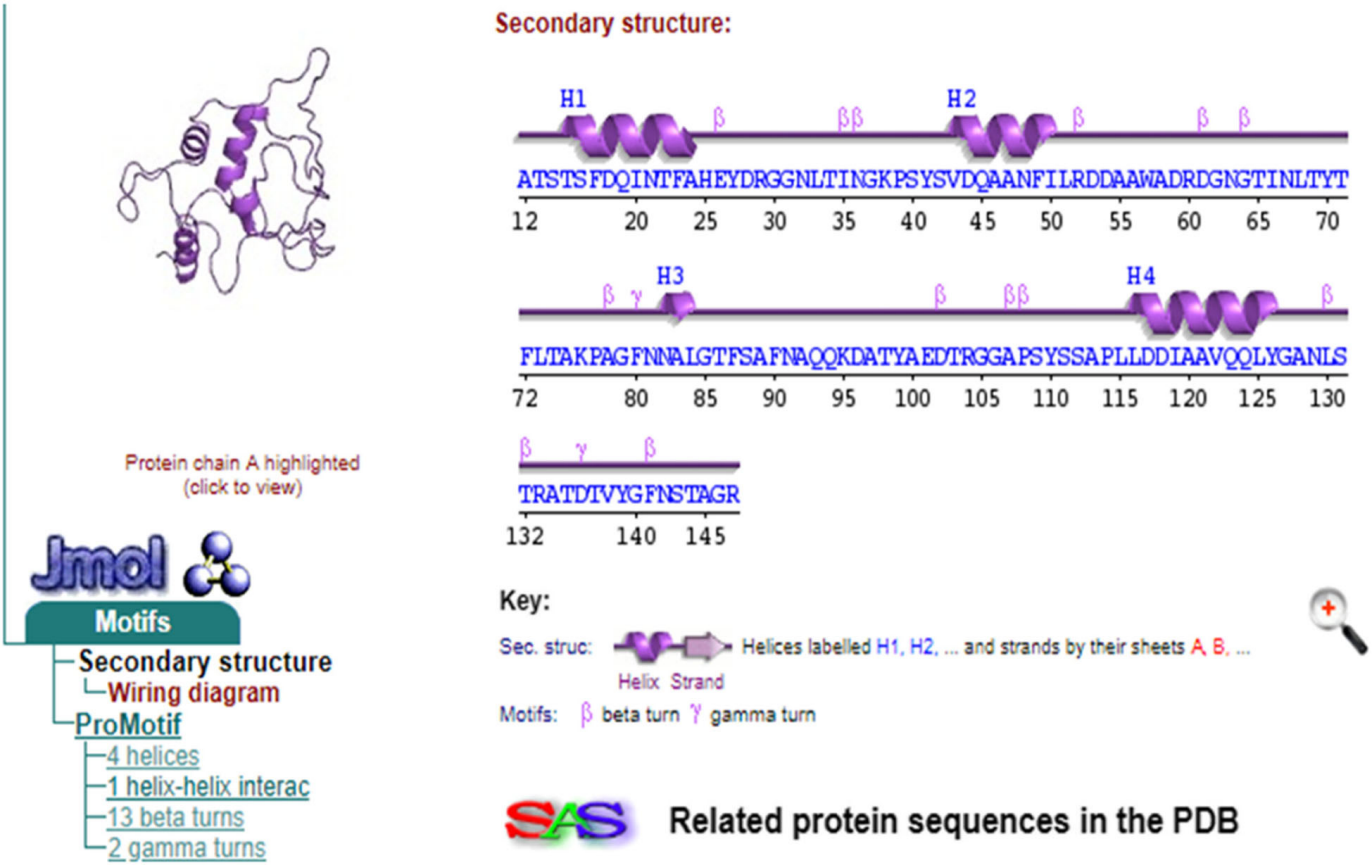
Secondary structure of S.AMP13 by ProFunc server where α-helices are labeled with H; beta and gamma turns are labeled with β and γ

**Fig. 17:**
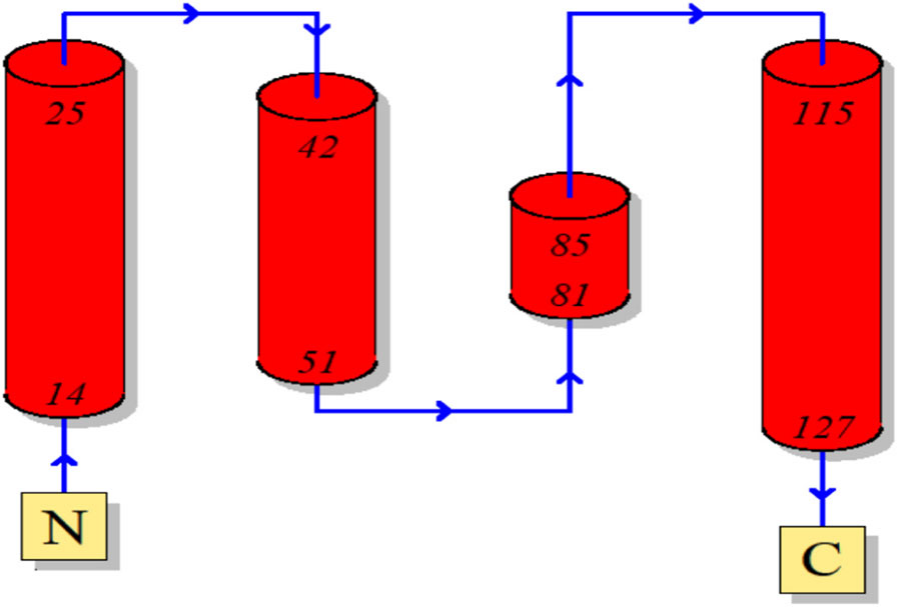
The topology diagram of the structural domain in S.AMP13. The diagram shows the relative location of the α-helices represented by the red cylinders, and the blue color arrow indicates the directionality of the protein chain from the N- to C-terminal

**Fig. 18:**
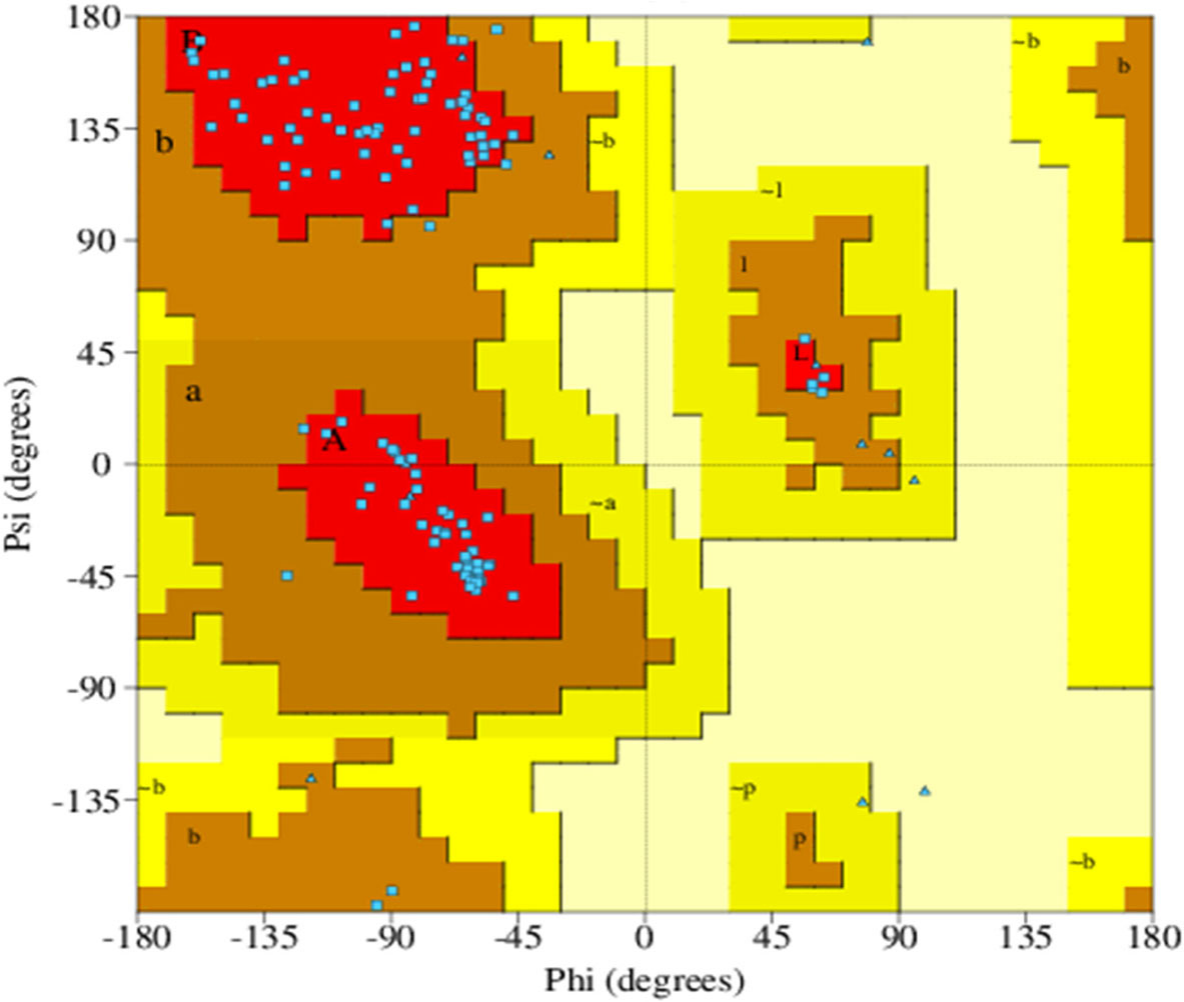
Ramachandran plot of S.AMP 13 using PROCHECK

Protein structure was predicted by homology modeling using a server like “SWISS-MODEL.” Sequence analysis revealed that S.AMP13 showed a maximum percent identification (55.88%) and sequence coverage with the alkaline metalloprotease template [1jiw.1.A]. Therefore, it was employed as a template for the 3D model prediction of S.AMP13 as shown in Fig. 14, and the model was also visualized by using PyMol (Fig. 15). The PDBsum of ProFunc server provided the secondary structure of the designed protein which contains 4-helical structures, 1 helix-helix interaction, 13 β turns, and 2 γ turns as shown in Fig. 16. It revealed that the S.AMP 13 enzyme contained 21.3% of α-helix, 76.5% of other structure (coil), and 2.2% 3–10 helices. The PROCHECK of the PDBsum server was employed for the evaluation of the stereochemical quality of the designed structure S.AMP13. The topology diagram of the structural domain in S.AMP13 is shown in Fig. 17. Ramachandran plot for the model was shown in Fig. 18 and revealed that 91.5% of amino acid residues were in the most favored region represented by red patches, 8.5% of amino acid residues were in the additionally allowed region represented by yellow fields, and there were no residues located in the disallowed regions which are represented by white field. Assessment of Ramachandran plot analysis confirms that the generated model was good in quality.

## Discussion

Serratiopeptidase is an alkaline metalloprotease produced from various sources such as *Serratia marcescens*, *Pseudomonas aeruginosa*, *Proteus mirabilis*, *Erwinia chrysanthemi* [4, 5], *Xenorhabdus* [6, 7], *Deinococcus radiodurans* [8], and *Bacillus subtilis* [9]. In this present study, the protease of *S. hydrogenans* var. MGS13 was purified from the optimized medium by following a two-step procedure. In the first step, the supernatant was precipitated with ammonium sulfate followed by gel filtration using Sephadex G-100 (Sigma Aldrich). After gel filtration, the purity of the enzyme was tested with SDS-PAGE, and a single band of 32 kDa was obtained. The molecular mass of the purified metalloprotease obtained from *S. hydrogenans* var. MGS13 was quite close to the molecular mass of alkaline metalloprotease from *Pseudomonas aeruginosa* MN1 [24] with a molecular weight of 32 kDa. Moreover, this signifies the fact that the mass of the purified enzyme was lower when compared to alkaline metalloprotease obtained from *Serratia marcescens* [25] and *Xenorhabdus indica* [7], where the reported molecular weight was between 46 and 60 kDa. This result illustrates that the molecular mass of purified protease obtained from the *S. hydrogenans* var. MGS13 was not similar with serratiapeptidase obtained from *Serratia marcescens* and shows more similarity with serralysin-like alkaline metalloprotease from *Pseudomonas*. Casein zymogram showed a single hydrolytic band which confirmed that the purified enzyme was a protease. Similar results have been observed in the case of serratiopeptidase obtained from *Serratia marcescens* [25] and fibrinolytic metalloprotease from *Bacillus cereus* B80 [26].

The ideal pH for the serratiopeptidase activity was found to be pH 9.0 which is similar to the serratiopeptidase obtained from *S. marcescens* [25] and strongly indicates that the enzyme is alkaline in nature. However, alkaline metalloprotease obtained from *Pseudomonas aeruginosa* MN1 showed a slight variation where the optimum pH for the enzyme activity was 8.0 [24]. The purified serratiopeptidase showed a maximum stability between pH 7 and 9, whereas the serratiopeptidase from *Serratia sp*. RSPB11 [25] showed 50% of activity at pH 6.0–10.0 and a fibrinolytic metalloprotease from *Bacillus cereus* B80 [26] showed maximum stability at pH 6.0–9.0. These observations indicate the variation existing in the stability of peptidases over different pH ranges.

The temperature effect on enzyme activity highlights the fact that the reaction environment temperature regulates the serratiopeptidase activity, and it requires an ideal temperature for cleaving the substrate. Similar results were observed in case of alkaline metalloprotease from *Serratia* sp. RSPB1 [25], *Bacillus brevis* MWB-01 [27], and *Pseudomonas fluorescens* 114 [28], where optimum enzyme activity was observed at 37 °C, 40 °C, and 35 °C, respectively. In the thermal stability studies, more than 50% residual activity was noticed at 60 °C, revealing that the protease of *S. hydrogenans* var. MGS13 is moderately thermostable. Similarly, serratiopeptidase obtained from *S. marcescens* [25] and alkaline metalloprotease obtained from *Bacillus cereus* B80 [26] retained 50% of their activities at 50 °C and 70 °C, respectively.

The impact of different inhibitors on the enzyme activity was evaluated to know the nature of purified protease of *S. hydrogenans* var. MGS13. PMSF at 1, 5, and 10 mM had little effect on serratiopeptidase activity confirming that the enzyme is not a serine protease, because serine proteases are strongly inactivated by PMSF at a concentration ranging from 0.1 to 1 mM [29]. However, the activity of purified protease was impaired by EDTA indicating that metal ion plays an indispensable role in the catalytic action of an enzyme which is conclusively indicating that this enzyme comes under metalloproteases class. The low inactivation rate caused by 1 mM EDTA (55%) might have been caused by competition among the excess of metals present at non-active sites. As EDTA concentration increases, the inhibitory effect is also increased, and the maximum inhibition was noticed with 10 mM EDTA. The study was also conducted by casein clearing zone method [22] in order to check the inhibitory effect on the enzyme activity visually. This method allows accuracy, and the hydrolytic zone produced on casein agar could be related to the amount of protease in the sample. The enzyme was strongly inactivated by EDTA than PMSF, thereby a small hydrolytic zone was observed in the presence of EDTA indicating that the purified enzyme is a metalloprotease. This procedure is more desirable for evaluating the change in activity and determines the nature of protease. Further, the increase in activity was also noticed in the presence of Zn^2+^, Na^+^, and K^+^ which illustrates that serratiopeptidase activity was positively regulated by these metal ions at 5 mM concentration. However, the presence of metals like Mg^2+^ and Cu^2+^ in the reaction mixture retarded the catalytic activity which is in contrast with serratiopeptidase from *Serratia marscens* where Mg^2+^ and Cu^2+^ enhanced the activity. This kind of variation in activity with the presence of a specific metal ion was also noticed with metalloprotease from *Serratia marscens* [25] where Ca^2+^, Co^2+^, Cu^2+^, K^+^, Mg^2+^, Na^+^, and Zn^2+^ increased the enzyme activity. Similar results have also been observed with the protease from *Bacillus brevis* MWB-01 [27] where the addition of Ca^+2^ and Mn^+2^ enhanced the enzyme activity whereas Hg^2+^ and Zn^2+^ retarded the activity.

The specific metal ions such as Ca^2+^, Zn^2+^, Na^+^, and K^+^ have significantly regained the metalloprotease activity, and the results were similar with alkaline metalloprotease of *Serratia marcescens* and *Pseudomonas fluorescens* [30] where Ca^2+^and Zn^2+^were effective in regaining the enzyme activity of EDTA inactivated metalloproteases.

The enzyme was excised from the SDS-PAGE gel as a single band and analyzed by LC-MS/MS for peptide mapping. The identification was done using the whole Swiss-Prot database for the species of interest (*Streptomyces*) and alkaline metalloprotease protein in different species to check the presence of different proteins in the sample with a false determination rate of 4%. The obtained partial amino acid sequence showed the highest homology with an alkaline metalloendoprotease of *Pseudomonas syringae pv. maculicola* (33769 mol. wt). Similarly, partial amino acid sequence of alkaline metalloprotease obtained from the *Pseudomonas aeruginosa* showed the highest homology with serralysin protease [30] and in another study, peptide fragments of X*enorhabdus nematophila* showed the highest homology with alkaline metalloprotease of *Pseudomonas aeruginosa* [31]*.* Phylogenetic analysis revealed that S.AMP13 showed the closest sequence similarity with alkaline metalloprotease of *Pseudomonas* sp. and in a similar manner, alkaline metalloprotease of *Pseudomonas* sp. showed sequence similarity with serralysin from *Serratia marcescens* [32]. The presence of peptide sequence ANLSTRATDTVYGFNSTAGR in the conserved region indicates that this enzyme (S.AMP 13) might be as serralysin-like metalloprotease as shown in Figs. 12 and 13. Similarly, metalloprotease of various sources such as *P. aeruginosa*, *E. chrysanthemi*, and *S. marcescens* shares a common sequence pattern in the conserved region and can be grouped together as a serralysin family [30]. A 3D model of S.AMP13 was generated using homology modeling, and the quality of that model was ensured as the best model based on the number of residues in most favored and additionally allowed regions.

## Conclusions

The electrophoretic analysis strongly confirmed that the isolated enzyme was a protease, and molecular weight was ascertained around 32 kDa. Inhibitor studies and peptide mapping denoted that this enzyme S.AMP13 belongs to metalloprotease. This enzyme S.AMP13 shared a conserved region of serralysin at positions 128–147 in C-terminal which denotes that the enzyme belongs to the serralysin family. The 3D model of S.AMP13 protein structure was predicted by homology modeling method and structurally validated by PROCHECK server using the Ramachandran plot (Additional files 1 and 2). The present findings suggest that S.AMP13 obtained from *S. hydrogenans var*. MGS13 is a serralysin-like protease, and the properties of this enzyme makes it valuable for the development of anti-inflammatory agent.

## Additional files

Additional file 1: PROCHECK statistics: Ramachandran plot statistics. (PNG 28 kb)

Additional file 2: PROCHECK statistics: G-factors. (PNG 22 kb)

## Data Availability

All data generated or analyzed during this study are included in this published article.

## Abbreviations

ACN: Acetonitrile
BLAST: Basic Local Alignment Search Tool
CDD: Conserved Domain Database
ClustalX: Clustal with a graphical user interface
D: Dimensional
DTT: Dithiothreitol
EDTA: Ethylene diamine tetra acetic acid
FA: Formic acid
FASTA: Pairwise Alignment Program
HGF: Horse gram flour
IP: Indian Pharmacopoeia
LC-MS: Liquid chromatography and mass spectrometry
MEGA: Molecular Evolutionary Genetic Analysis
NCBI: National Center for Biotechnology Information
PDB: Protein Data Bank
PLGS: ProteinLynx Global SERVER
PMSF: Phenyl methyl sulphonyl fluoride
QMEAN: Qualitative model energy analysis
Q-TOF: Quadrupole time-of-flight
S.AMP13: Alkaline metalloprotease obtained from *S. hydrogenans* var. MGS13
SDS: Sodium dodecyl sulfate
SMTL: SWISS-MODEL Template Library
SSF: Solid-state fermentation
SD: Standard deviation
SWISS: Structural Bioinformatics Web Server
UPLC: Ultra-pressure liquid chromatography
var.: Variant
β: Beta
γ: Gamma
δ: Delta

## References

[1] Rawlings ND, Barrett AJ, Bateman A (2012). MERPOS: the database of proteolytic enzymes, their substrates and inhibitors. Nucleic Acids Res..

[2] Al-Khateeb TH, Nusair Y (2008). Effect of the proteolytic enzyme serrapeptase on swelling, pain and trismus after surgical extraction of mandibular third molars. Int J Oral Maxillofac Surg..

[3] Russell LW, Judith SB (1990). Phe5 (4-nitro)-bradykinin: a chemogenic substrate for assay and kinetics of the metalloendopeptidase meprin: Anal. Biochem..

[4] Lakshmi Bhargavi P, Prakasham RS (2012). Proteolytic enzyme production by isolated by isolated *Serratia* sp RSPB11: role of environmental parameters. Curr Trends Biotechnol Pharm..

[5] Wu D, Ran T, Wang W, Xu D (2016). Structure of a thermo stable serralysin from *Serratia* sp. FS14 at 1.1A resolution. Acta Cryst.

[6] Massaoud MK, Marokhazi J, Venekei I (2011). Enzymatic characterization of a seralysin-like metalloproteases from the entomopathogen bacterium, *Xenorhabdus*. Biochimic Biophys Acta..

[7] Pranaw K, Surender S, Debjani D, Nipendra S, Garima S, Sudershan G (2013). Extracellular novel metalloprotease from *Xenorhabdus indica* and its potential as an insecticidal agent. J Microbiol Biotechnol.

[8] Basu B, Apte SK (1784). A novel serralysin metalloprotease from *Deinococcus radiodurans*. Biochem Biophys Acta..

[9] Kyostio SR, Cramer CL, Lacy GH (1991). Erwinia carotovora subsp. Carotovora extracellular protease; characterization and nucleotide sequences of the gene. J. Bacteriol..

[10] MacCabe AP, Polaina J. (2007) Industrial enzymes: structure, function and applications 1st ed. Springer Dordrecht: Netherlands.

[11] Kreger AS, Griffin OK (1975). Cornea-damaging proteases of *Serrratia marcescens*. Invest Ophthalmol..

[12] Wang J, Wang M, Wang Y (1998). Purification and characterization of novel fibrinolytic enzymes from *Streptomyces* sp. Chin J Biotechnol..

[13] Uesugi Y, Usuki H, Iwabuchi M, Hatanaka T (2011). Highly potent fibrinolytic serine protease from *Streptomyces*. Enzyme Microb Technol..

[14] Simkhada JR, Mander P, Cho SS, Yoo JC (2015). A novel fibrinolytic protease from *Streptomyces* sp.CS684. Process Biochem..

[15] Jyothi V, Girija Sankar G, Prabhakar T (2014). Isolation of novel mutant strain for enhanced production of extracellular serratiopeptidase from mangrove soil. Int J Pharma Sci Rev Res..

[16] Jyothi V, Haritha C, Girija Sankar G, Prabhakar T (2014). Application of response surface methodology in medium components optimization to enhance serratiopeptidase production by *S. hydrogenans* MGS13. Eur Sci J.

[17] Deutzmann R (2004). Structural characterization of proteins and peptides. Methods Mol Med..

[18] Indian Pharmacopoeial Commission (2010). Indian pharmacopoeia.

[19] Lowry OH, Rosebrough NJ, Farr AL, Randall RJ (1951). Protein measurement with Folin reagent. J Biol Chem..

[20] Laemmli UK (1970). Cleavage of structural proteins during the assembly of head of bacteriophage T4. Nature..

[21] Garcia-Carreno FL, Dimes LE, Haard NF (1993). Substrate gel electrophoresis for composition and molecular weight of proteinase or proteinaceous proteinase inhibitors. Anal Biochem..

[22] Katsushi Y, Naomi F, Seiko T (1988). Application of casein agar plate method for determination of protease activity. J J Toxicol Environ Health..

[23] Larkin MA, Blackshields G, Brown NP, Chenna R, McGettigan PA (2007). Clustal W and Clustal X version 2.0. Bioinformatics..

[24] Bayoudh A, Gharsallah N, Chamkha M, Dhouib A, Ammar S, Nasri M (2000). Purification and characterization of an alkaline protease from *Pseudomonas aeruginosa* MN. J Ind Microbiol Biotech..

[25] Bhargavi PL, Prakasham RS (2013). A fibrinolytic, alkaline and thermo stable metalloprotease from the newly isolated *Serratia* sp. RSPB11. Int J Biol Macromol..

[26] Rajasree S, Rajani S. MALDI-TOF MS and CD spectral analysis for identification and structural prediction of a purified, novel, organic solvent stable, fibrinolytic metalloprotease from *Bacillus cereus* B80.Bio. Med. Res. Int. 2015; Article ID 527015,1-13. 10.1155/2015/527015 PMC4352737 25802851

[27] Olajuyigbe FM, Faladel MA (2014). Purification and partial characterization of serine alkaline metalloprotease from *Bacillus brevis* MWB- 01. Bioresour. Bioprocess..

[28] Hamamoto T, Kaneda M, Horikoshi K, Toshiaki K (1994). Characterization of a protease from a psychrotroph, Pseudomonas fluorescens 114. Appl Environ Microbiol..

[29] Salarizadeh N, Hasannia S, Noghabi NA, Sajedi RH (2014). Purification and characterization of 50 kDa extracellular metalloprotease from *Serratia* sp. ZF03. Iran J Biotech.

[30] Kim HJ, Tamanoue Y, Jeohn GH, Iwamatsu A (1997). Purification and characterization of an extracellular metalloprotease from *Pseudomonas fluorescens*. J Biochem.

[31] Caldas C, Cherqui A, Pereira A, Simoes N (2002). Purification and characterization of an extracellular protease from *Xenorhabdus nematophila* involved in insect immunosuppressant. Appl Environ Microbiol..

[32] Shanks RM, Stella NA, Hunt KM, Brothers KM, Zhang L, Thibodeau PH (2015). Identification of SlpB, a cytotoxic protease from *Serratia marcescens*. Infect Immune.

