# Supplementary figures and images for: Purification, characterization, and structural elucidation of serralysin-like alkaline metalloprotease from a novel source
Source: J Genet Eng Biotechnol. 2019 Sep 23;17:1. doi: 10.1186/s43141-019-0002-7 (PMC6821148; doi:10.1186/s43141-019-0002-7)

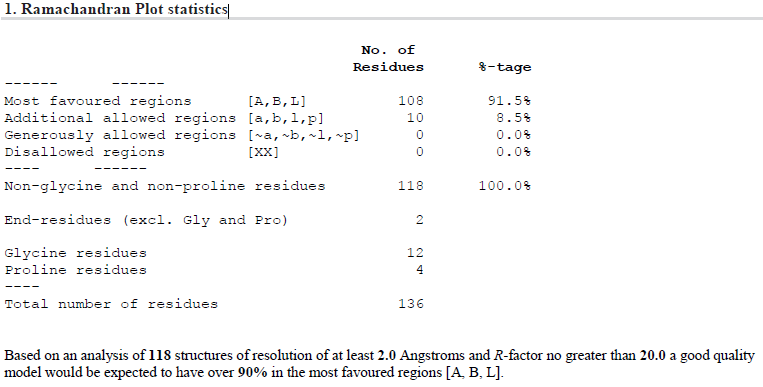

Supplement: Supplementary file 1 — PROCHECK statistics: Ramachandran plot statistics. (PNG 28 kb) [file 43141_2019_2_MOESM1_ESM.png]

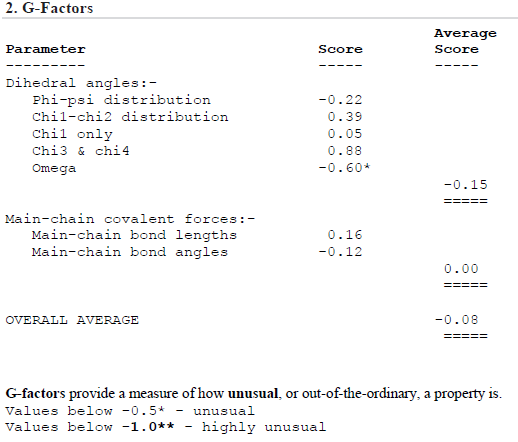

Supplement: Supplementary file 2 — PROCHECK statistics: G-factors. (PNG 22 kb) [file 43141_2019_2_MOESM2_ESM.png]
